# Overexpression of Global Regulator Talae1 Leads to the Discovery of New Antifungal Polyketides From Endophytic Fungus *Trichoderma afroharzianum*

**DOI:** 10.3389/fmicb.2020.622785

**Published:** 2020-12-23

**Authors:** Zhuang Ding, Xiao Wang, Fan-Dong Kong, Hui-Ming Huang, Yan-Na Zhao, Min Liu, Zheng-Ping Wang, Jun Han

**Affiliations:** ^1^Institute of BioPharmaceutical Research, Liaocheng University, Liaocheng, China; ^2^Hainan Key Laboratory for Research and Development of Natural Product From Li Folk Medicine, Institute of Tropical Bioscience and Biotechnology, Chinese Academy of Tropical Agriculture Sciences, Haikou, China; ^3^School of Life Sciences, Liaocheng University, Liaocheng, China

**Keywords:** global regulator, LaeA, polyketides, antifungal activity, endophytic fungi, Trichoderma afroharzianum

## Abstract

Transcription regulation caused by global regulators exerts important effects on fungal secondary metabolism. By overexpression of the global regulator Talae1 in a *Ficus elastica*-associated fungus *Trichoderma afroharzianum*, two structurally new polyketides (**1** and **2**) that were newly produced in the transformant were isolated and identified. Their structures, including the absolute configurations, were elucidated through a combination of high resolution mass spectrometer (HRMS), NMR, and electronic circular dichroism (ECD) calculations. The growth inhibitory activities of compounds **1** and **2** were evaluated against four bacteria and six plant-pathogenic fungi. Compound **1** showed the highest antifungal activity against *Botrytis cinerea* and *Fusarium oxysporum* f. sp. *nicotianae* with MIC of 8 μg/ml. To the best of our knowledge, this is the first study to report on the application of the global regulator in *T. afroharzianum* to activate the biosynthesis of bioactive secondary metabolites.

## Introduction

The genus *Trichoderma* is a ubiquitous fungal group comprising of more than 340 species (Zeng and Zhuang, 2019). Some *Trichoderma* species are widely used in agriculture as biological control agents to prevent the growth of other phytopathogenic fungi and to promote the development of crop plants (Benítez et al., 2004; Reino et al., 2008). The genus *Trichoderma* has been reported to be an excellent producer of bioactivity secondary metabolites (SMs). Several hundreds of SMs, including polyketides, non-ribosomal peptides, terpenoids, and alkaloids, have been identified and isolated in *Trichoderma* species and strains from different environments (Reino et al., 2008; Degenkolb et al., 2015; Li et al., 2019). Some of the SMs are considered to play a significant role in stimulating plant growth or providing defense against plant pathogens (Vinale et al., 2008).

Recent genome sequencing of many fungal species has revealed that most of the biosynthetic gene clusters (BGCs) are cryptic or lowly expressed under general culture conditions (Rutledge and Challis, 2015; Wu et al., 2020). Similarly, although diverse metabolites have been discovered from the genus *Trichoderma*, genome sequencing has revealed that there are more BGCs than we have discovered (Mukherjee et al., 2013; Zeilinger et al., 2016), suggesting that the potential of *Trichoderma* fungi to produce more undetected metabolites has hidden and needs to be activated.

To activate the cryptic biosynthetic potential and reveal more SMs, several strategies, such as ribosome engineering, transcriptional regulation, epigenetic perturbation, and heterologous expression, have been developed (Wiemann and Keller, 2014; Wu et al., 2016). Besides, the manipulation of global transcriptional regulators is also reported to be a feasible strategy for the activation of cryptic BGCs. The LaeA protein was first discovered as a global regulator of secondary metabolism in *Aspergillus nidulans* (Bok and Keller, 2004). Because of sequence similarity to histone methyltransferases, LaeA may achieve global transcriptional regulation by influencing chromatin modification (Brakhage, 2013). Reyes-Dominguez et al. (2010) reported that LaeA reversed the repressive chromatin structure caused by some negative regulators, resulting in the activation of BGCs in *A. nidulans*. Furthermore, LaeA is conserved in filamentous fungi, and thus its gene homologs are found in other filamentous fungi including *Aspergillus carbonarius* (Linde et al., 2016), *Aspergillus fumisynnematus* (Hong et al., 2015), *Alternaria alternata* (Estiarte et al., 2016), *Fusarium verticillioides* (Butchko et al., 2012), *Monascus ruber* (Liu et al., 2016), and *Penicillium chrysogenum* (Kosalková et al., 2009) and *Penicillium brocae* (Wang et al., 2020). Numerous studies have also determined that the presence of LaeA facilitates the expression of multiple BGCs. For instance, Jiang et al. (2016) reported that overexpression of *laeA* in *Chaetomium globosum* upregulated the expression of chaetoglobosin BGC and led to the discovery of a new cytochalasan. Yu et al. (2019) reported the discovery of a series of sorbicillinoids including two new ones by overexpression of laeA in *Penicillium dipodomyis*. Thus, overexpression of LaeA is considered as a useful strategy in activating silent biosynthetic pathways and promoting the discovery of novel SMs in fungi.

Endophytic fungi are increasingly recognized as a significant reservoir of bioactive metabolites (Chandra, 2012; Wu et al., 2016). Due to the absence of a simulated ecological environment, the BGCs of endophytic fungi are not effectively expressed under standard laboratory conditions, thus reducing the probability of detecting new SMs (Mao et al., 2015). In our previous research work, the endophytic fungus *Trichoderma afroharzianum* Fes1712 isolated from rubber tree *Ficus elastica* was shown to produce novel isocoumarin analogues (Ding et al., 2019). To further activate its chemical potential, we constructed a *laeA*-like gene (*talae1*) overexpression transformant of *T. afroharzianum* and investigated the effect of *laeA*-like gene overexpression on secondary metabolic profile. Chemical analysis of SMs produced by the overexpression strain led to the discovery of two new polyketides (**1**, **2**). In this study, structural elucidation and biological evaluation of the two new compounds are presented.

## Materials and Methods

### General Experimental Procedures

DNA restriction enzymes were purchased from Transgen Biotech Co., LTD (Beijing, China). Polymerase chain reaction (PCR) was performed using TransStart® Fastpfu Fly DNA Polymerase (Transgen Biotech, Beijing, China). Optical rotations were measured on a P-1020 digital polarimeter (JASCO Corporation, Tokyo, Japan). ECD spectra were recorded on a JASCO J-815 spectropolarimeter (JASCO Corporation). UV spectra were recorded on Waters 2487 (Waters Corporation, Milford, MA, United States). HRESIMS and ESIMS spectra were measured on Thermo Scientific LTQ Orbitrap XL mass spectrometer (Thermo Fisher Scientific). NMR spectra were recorded on Agilent 500 MHz DD2 spectrometer (Agilent Technologies Inc., Santa Clara, CA, United States). Semi-preparative HPLC was performed using a YMC Pack ODS-A column (250 × 10 mm, 5 μm, 3 ml/min, YMC Co., Ltd., Kyoto, Japan). Column chromatography was performed on silica gel (200–300 mesh, Qingdao Marine Chemical Inc., Qingdao, China) and Sephadex LH-20 (GE Healthcare, Uppsala, Sweden).

### Fungal Material

The fungal wild strain Fes1712 (WT) was originally isolated from fresh leaves of *F. elastica* collected from Liaocheng University Arboretum, Liaocheng, Shandong Province of China and identified as *T. afroharzianum* based on *tef1* and *rpb2* sequences (Chaverri et al., 2015; Supplementary Figure S1).

### Generation of the OE::Talae1 Strain

Because the genomic sequence of *T. afroharzianum* Fes1712 is currently unknown, the genomic sequence of *Trichoderma harzianum* CBS 226.95 was used for gene selection and initial primer design. A *laeA*-like gene was identified using Local-Blast with *A. nidulans* LaeA gene (AN0807) as the query. The two pairs of specific primers, including two inner primers (Ta85012iF/R) with terminal XbaI and EcoRV restriction sites, were used as primers of the nested-PCR amplification of *talae1* from genomic DNA of strain Fes1712. The PCR product of the *talae1* gene was digested using endonucleases and inserted into the same restriction site of the pZeo vector (Tang et al., 2017) to create pZeo-talae1 (Supplementary Figure S2). The *talae1* gene from strain Fes1712 was sequenced by Sangon Biotech (Shanghai, China) and deposited in the GenBank Database under the accession numbers MT313929. The overexpression vector pZeo-talae1 comprised of a continuous expression cassette, including the PgpdA promoter, *talae1* gene, and TtrpC terminator, and two selection markers (ampicillin resistance gene for selection of *Escherichia coli* strain and bleomycin resistance gene for the Fes1712 transformant). The recombinant vector was transformed into *E. coli* strain Trans1-T1 to extract plasmids for transformation. The extracted plasmids were then transferred into WT yielding a transformant with overexpressed *talae1* gene (*OE::Talae1*) by PEG-mediated protoplast transformation (Tang et al., 2017), while the control transformant was also generated using a vacant pZeo. The transformants were selected and purified in the presence of bleomycin. The genotype of the overexpression transformant was confirmed by diagnostic PCR (Supplementary Figure S3). The oligonucleotide sequences for PCR primers are presented in Supplementary Table S1.

### Culture, Fermentation, and Extraction

For SMs production, the overexpression transformant and control transformant were cultured on PDA medium (PDA, 20% potato, 2% dextrose, and 1.5% agar) at 28°C for 5 days. They were then inoculated into 1 L Erlenmeyer flasks, each containing 80 g of rice and 120 ml distilled water and incubated at 28°C under static conditions. After 15 days, the fermented substrate in each flask was broken using a disperser (T18, IKA, Germany) and extracted three times with 200 ml MeOH. The liquid layers were collected and evaporated to remove MeOH. The residual extract was suspended in H_2_O and extracted three times with an equal volume of EtOAc. The EtOAc layers were separated and evaporated under reduced pressure to yield the solid extract.

### Purification

The extract from the overexpression transformant (6.7 g) was applied on silica gel using a step gradient elution with petroleum ether−EtOAc (*v*/*v* 10:1, 5:1, 2:1, 1:1, 1:3) and EtOAc−MeOH (*v*/*v* 10:1, 3:1, 1:1, 0:1) to give nine fractions (Fr. 1–9). Fraction 7 was fractionated on MPLC (60–100% MeOH/H_2_O, 45 min) to give five fractions (Fr. 7.1–7.5). Fraction 7.3 and Fraction 7.4 were further purified by semipreparative HPLC eluting with 70% MeOH−H_2_O to obtain compound **1** (9.0 mg) and **2** (4.5 mg).

Compound **1**: white amorphous powder; [*α*]^20^_D_ − 36.1 (*c* 0.10, MeOH); ECD (MeOH) *λ*_max_ (∆*ε*) 304 (+2.01), 233 (−10.55) nm; UV (MeOH) *λ*_max_ (log *ε*) 299 (2.01), 210 (3.14) nm; positive HR-ESI-MS (*m/z*): 263.1282 [M + H]^+^ (calcd. for C_15_H_19_O_4_, 263.1283; Supplementary Figure S5); ^1^H and ^13^C NMR data, see Table 1 and Supplementary Figures S6–S11.
Compound **2**: white amorphous powder; [α]^20^_D_ − 44.2 (*c* 0.10, MeOH); ECD (MeOH) *λ*_max_ (∆ε) 304 (+2.52), 235 (−9.74) nm; UV (MeOH) *λ*_max_ (log *ε*) 300 (2.10), 210 (3.15) nm; positive HR-ESI-MS (*m/z*): 277.1435 [M + H]^+^ (calcd. for C_16_H_21_O_4_, 277.1439; Supplementary Figure S12); ^1^H and ^13^C NMR data, see Table 1 and Supplementary Figures S13–S18.

**Table 1:** ^1^H and ^13^C NMR data of the compounds **1** and **2** (500 MHz in CD_3_OD); *δ* in p.p.m., *J* in Hz.

Position	Compound (**1**)		Compound (**2**)	
	*δ*_C_, type	*δ*_H_, (*J* in Hz)	*δ*_C_, type	*δ*_H_, (*J* in Hz)
1	106.8, CH	6.29, *s*	106.6, CH	6.35, *s*
2	154.0, Cq		154.0, Cq	
3	111.8, Cq		112.0, Cq	
4	153.5, Cq		153.6, Cq	
5	114.9, Cq		115.3, Cq	
6	134.1, Cq		131.6, Cq	
7	76.6, CH	5.33, *s*	85.7, CH	4.98, *s*
8	199.1, Cq		197.6, Cq	
9	122.6, CH	6.12, *d*, (15.3)	122.7, CH	6.17, *d*, (15.3)
10	143.5, CH	7.24, *dd*, (15.3, 10.5)	143.6, CH	7.23, *dd*, (15.3, 10.6)
11	130.0, CH	6.21, *dd*, (15.5, 10.5)	130.0, CH	6.21, *dd*, (15.5, 10.6)
12	141.3, CH	6.25, *dq*, (15.5, 6.4)	141.3, CH	6.25, *dq*, (15.5, 6.2)
13	17.4, CH_3_	1.81, *d*, (6.4)	17.4, CH_3_	1.82, *d*, (6.2)
14	7.6, CH_3_	2.06, *s*	7.6, CH_3_	2.06, *s*
15	10.2, CH_3_	2.16, *s*	10.3, CH_3_	2.13, *s*
7-OCH_3_			55.6, CH_3_	3.30, *s*

### Bioactivity Assay

Antimicrobial activities of the isolated compounds were evaluated against four bacteria (*Bacillus subtilis* CMCC 63501, *E. coli* CMCC 44102, *Pseudomonas aeruginosa* CMCC 10104, and *Staphylococcus aureus* CMCC 26003) and six plant-pathogenic fungi (*A. alternata* ACCC 36110, *Botrytis cinerea* ACCC 36028, *Colletotrichum lagenarium* ACCC 30016, *Fusarium oxysporum* f. sp. *nicotianae* TRICAAS 0101, *Gaeumannomyces graminis* var. *graminis* TRICAAS 0191, and *Thielaviopsis basicola* TRICAAS 0207) using the 96-well plate microdilution method (Zhang et al., 2019). The phytopathogenic fungi were provided and deposited by the Tobacco Research Institute of the Chinese Academy of Agricultural Sciences, Qingdao, China. The tested compounds were prepared as 2-fold dilutions with DMSO and added (10 μl) to each well, containing the spore suspension (10^6^ CFU/ml, 10 μl) and culture medium (180 μl, Luria-Bertani medium for bacteria, potato dextrose medium for fungi), to obtain a final concentration of between 512 μg/ml to 1 μg/ml. After adequate mixing, the assay plates were incubated in the dark for 24–72 h at 30°C. The minimal inhibitory concentration (MIC) was determined as the lowest concentration at which no growth of pathogen was observed. Chloramphenicol and prochloraz were used as positive controls for antibacterial and antifungal assays, respectively. All tests were performed in triplicate.

## Results and Discussion

### Genome Mining and Overexpression of Talae1

**Figure 1:**
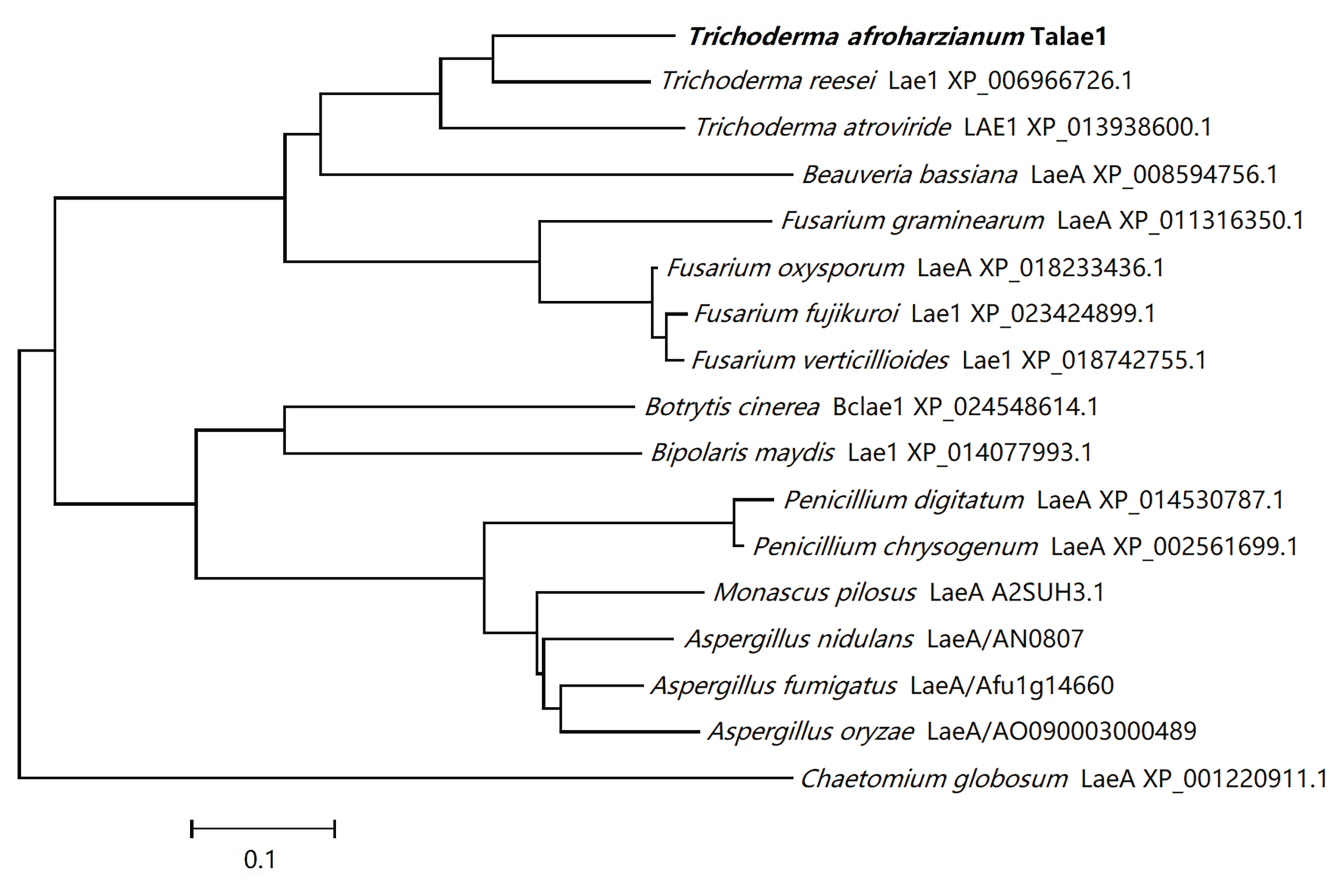
Phylogenetic tree analyses of Talae1 and its homologs from different species. Branch lengths are in proportion to distance.

A *laeA*-like gene was identified in the genomic sequence of *T. harzianum* CBS 226.95 using Local-blast. In the genomic sequence of *T. harzianum* CBS 226.95, the *laeA*-like gene is designated as M431DRAFT_85012 (DOE Joint Genome Institute). Subsequently, we designed special primers to clone *talae1* ORF from the genomic DNA of strain Fes1712 *via* nested-PCR. The obtained PCR fragment was 1,228 bp in size, and the predicted coding sequence was 999 bp, encoding a 332-residue polypeptide. Sequence analysis *via* InterProScan indicated that *talae1* protein was an S-adenosyl-L-methionine-dependent methyltransferase, which is consistent with the function of LaeA. BLAST analysis indicated that Talae1 protein had 73.9, 68.1, 57.1, and 56.8% sequence identity to Lae1 (XP_006966726.1) of *Trichoderma reesei* (Seiboth et al., 2012), Lae1 (XP_013938600.1) of *Trichoderma atroviride* (Aghcheh et al., 2013), LaeA (XP_018233436.1) of *F. oxysporum* (López-Berges et al., 2014), and Lae1 (XP_018742755.1) of *F. verticillioides* (Butchko et al., 2012), respectively (Figure 1).

**Figure 2:**
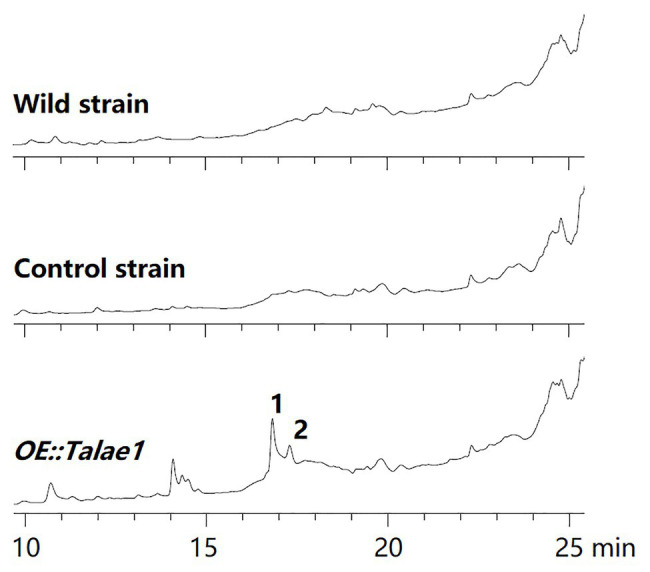
Comparative analysis of secondary metabolite HPLC profiles of *OE::Talae1* and control transformants. The numbers represent the compounds isolated from the *OE::Talae1* transformant. Detection was carried out at 210 nm.

**Figure 3:**
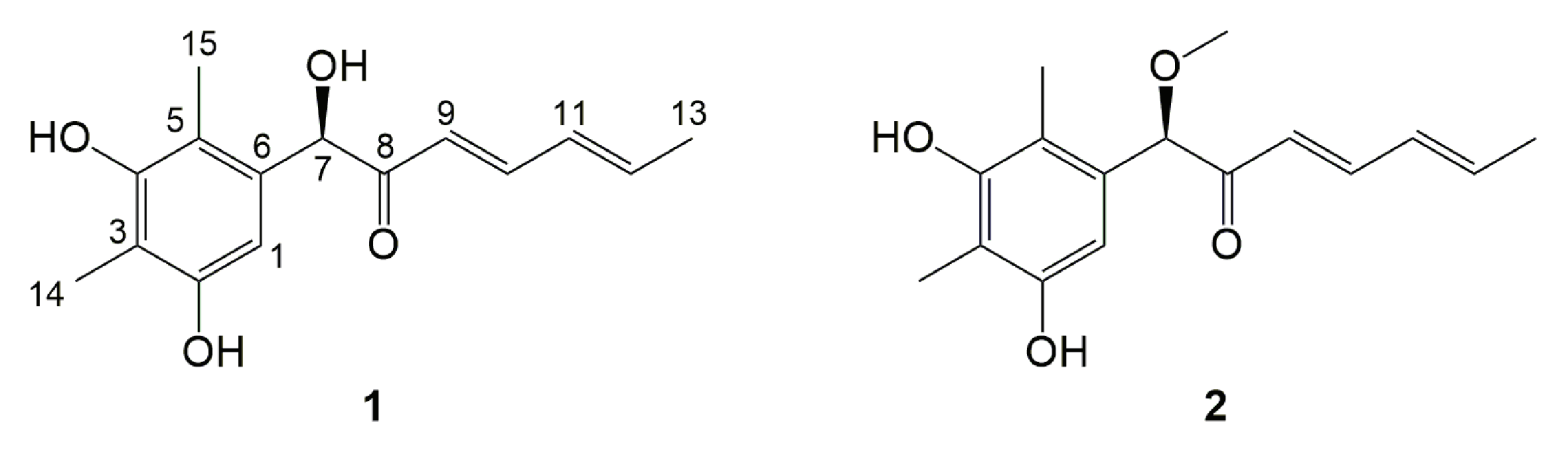
Structures of compounds **1** and **2** from the *OE::Talae1* transformant.

To examine the effect of *talae1* gene overexpression on secondary metabolism in strain Fes1712, *OE::Talae1* transformant and control transformant were constructed and cultured on rice medium at 28°C for 15 days under static conditions. Real time PCR was performed to determine the difference in the transcriptional levels of *talae1* gene between the *OE::Talae1* and control transformants. Results indicated that the transcriptional level of *talae1* gene was 7.7-fold upregulated in *OE::Talae1* transformant (Supplementary Figure S4). The fermentation products were extracted and analyzed using HPLC. *OE::Talae1* transformant showed an obvious change in the secondary metabolic profile, evidenced by the emergence of several new peaks (Figure 2), compared with that of the control transformant. Purification of the new peaks led to the isolation of two relatively high-yield compounds **1** and **2** (Figure 3).

### Structure Elucidation of the New Compounds

**Figure 4:**
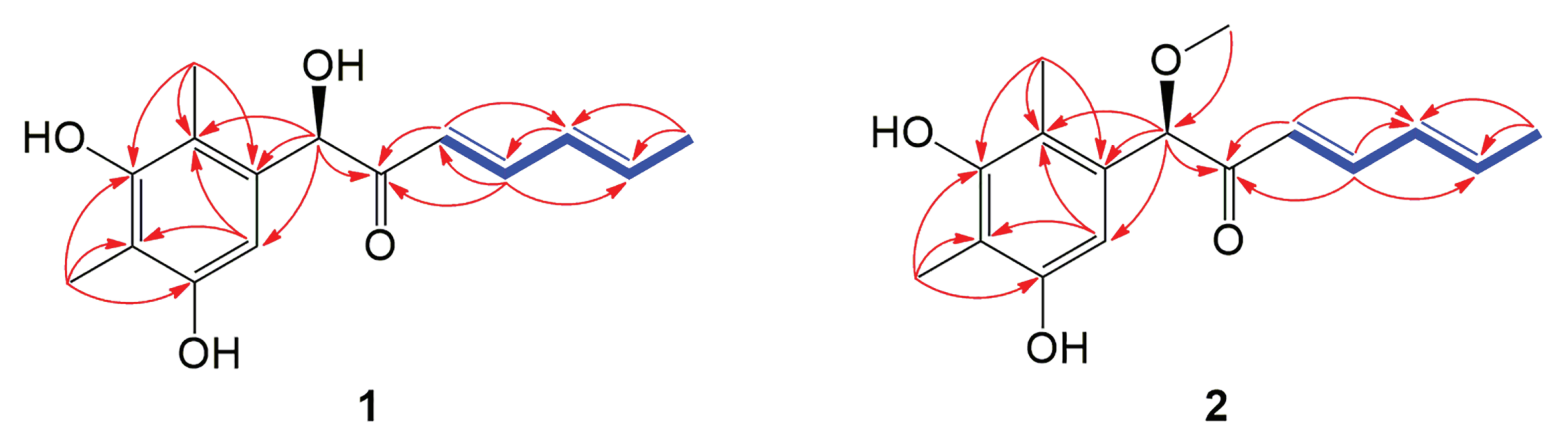
Key COSY (bold blue lines) and HMBC (red arrows) correlations of the compounds **1** and **2**.

**Figure 5:**
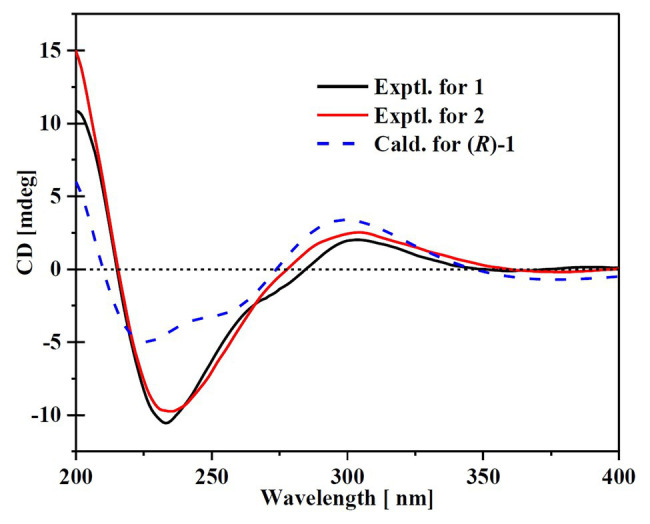
Experimental ECD of the compounds **1** and **2** and the calculated spectrum for (7*R*)**-1**.

Compound **1** was obtained as a white amorphous powder. The molecular formula C_15_H_18_O_4_ was determined by the positive HRESIMS *m/z* 263.1282 ([M + H]^+^, calcd. 263.1283), indicating 7 degrees of unsaturation. The ^1^H NMR spectrum of **1** revealed signals for five olefinic protons (*δ*_H_ 7.24, 6.29, 6.25, 6.21, and 6.12), an oxymethine (*δ*_H_ 5.33), and three methyl groups (*δ*_H_ 2.16, 2.06, and 1.81). While the ^13^C NMR, DEPT, and HSQC spectra exhibited the presence of 15 carbon resonance signals, including one conjugated ketone carbonyl (*δ*_C_ 199.1), nine olefinic or aromatic carbons with five protonated, one oxymethine (*δ*_C/H_ 76.6/5.33), and three methyls (*δ*_C/H_ 17.4/1.81, 10.2/2.16, and 7.6/2.06; Table 1). Analysis of the 2D NMR data of **1** revealed contiguous COSY correlations extending from H-9 to H-13, as well as heteronuclear multiple bond correlation (HMBC) correlation from H-10, H-9, and the oxymethine proton H-7 to C-8 carbonyl, indicating the presence of a 1-hydroxyhepta-3,5-dien-2-one side chain (Figure 4). The large *J* values of H-9/H-10 (*J* = 15.3 Hz) and H-11/H-12 (*J* = 15.5 Hz) revealed the *E*-configuration of the ∆^10^ and ∆^12^ double bonds (Figure 4). HMBC correlations from H_3_-15 to C-5, C-6, and the hydroxylated aromatic carbon C-4, from H_3_-14 to C-3 and the two hydroxylated aromatic carbons C-4 and C-2, and from the aromatic proton H-1 to C-3 and C-5 collectively led to the construction of the penta-substituted benzene core structure of compound **1** as shown in Figure 4. The presence of C-6/C-7 linkage between the penta-substituted benzene core structure and the 1-hydroxyhepta-3,5-dien-2-one side chain was demonstrated by HMBC correlations from H-7 to C-1, C-5, and C-6. To determine the absolute configuration of C-7 in **1**, the ECD spectrum of **1** was calculated using the time-dependent density functional theory (TD-DFT) at the B3LYP/6-31 + G (d) level using the Gaussian 09 program (Frisch et al., 2009). The result showed that the experimental ECD spectrum of **1** was in good agreement with the calculated ECD spectrum of (7*R*)-**1** (Figure 5), designating the absolute configuration of C-7 as *R*. Thus, compound **1** was determined to be (*R*,3*E*,5*E*)-1-(3,5-dihydroxy-2,4-dimethylphenyl)-1-hydroxyhepta-3,5-dien-2-one.

Compound **2** was isolated as a white amorphous powder with the molecular formula C_16_H_20_O_4_ determined based on HRESIMS (*m/z* 277.1413 [M + H]^+^), with an additional CH_3_ group compared to **1**. The 1D and 2D NMR data of compound **2** showed close similarity to those of **1**, with the only distinction attributable to the presence of a methoxy signal at *δ*_H/C_ 3.30/55.6 in **2** (Table 1). The key HMBC correlation from H_3_-16 (*δ* 3.67) to the oxymethine carbon C-7 (*δ* 172.1) indicated that the hydroxyl group at C-7 in **1** was methylated in **2**. The absolute configuration of C-7 in **2** was determined to be similar to that of **1** based on ECD spectra (Figure 5). Therefore, the structure of compound **2** was determined to be (*R*,*3E*,*5E*)-1-(3,5-dihydroxy-2,4-dimethylphenyl)-1-methoxyhepta-3,5-dien-2-one.

### Plausible Biogenetic Pathways Proposed for 1 and 2

**Figure 6:**
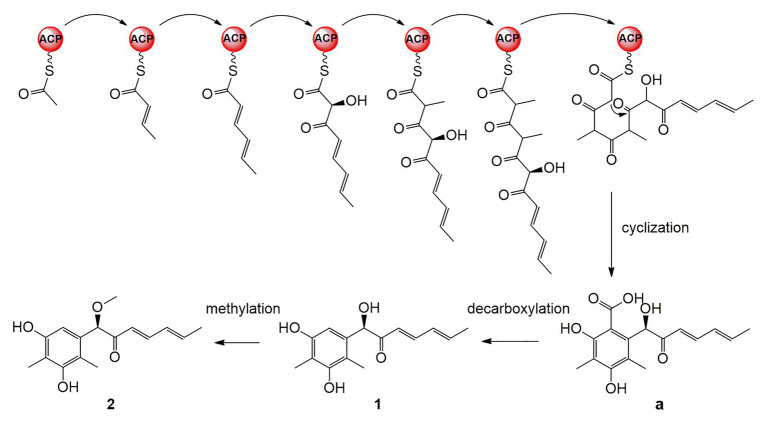
Plausible biosynthetic pathway for **1** and **2**.

A plausible biosynthetic pathway for **1** and **2** is proposed as shown in Figure 6. The polyketide chain primed with one acetyl-CoA starter unit and six malonyl-CoA extender units was selectively enolized, hydroxylated, methylated, cyclized, and finally released as the intermediate **a** (Wu et al., 2019). Compounds **1** and **2** are proposed to be generated by further decarboxylation and O-methylation (Shaw et al., 2015; Lebe and Cox, 2019).

### Bioactivity Assay

The newly produced compounds **1** and **2** were evaluated for antifungal activity against four bacteria and six plant-pathogenic fungi were tested. As shown in Table 2, compounds **1** and **2** showed selective antifungal activity with MIC values ranging from 8 to 32 μg/ml. Compound **1** displayed the highest growth inhibitory activity with MIC values of 8, 8, and 32 toward *B. cinerea*, *F. oxysporum* f. sp. *nicotianae*, and *C. lagenarium*, respectively. The genus *Trichoderma* has been shown to produce numerous polyketides with various bioactivities (Reino et al., 2008; Harned and Volp, 2011). For instance, fungal species of the *Trichoderma* genus are known to be the main producers of sorbicillin family (Harned and Volp, 2011; Derntl et al., 2017), which are structurally similar to the compounds **1** and **2**. Reátegui et al. (2006) have reported antifungal activity of sorbicillin analogues against *Aspergillus flavus* and *Fusarium verticillioides*. Besides, the compounds **1** and **2** also showed weak antibacterial activity against Gram-positive bacteria *S. aureus*.

**Table 2:** Antimicrobial activities of the compounds **1** and **2** (MIC, μg/ml).

Compound	Bacteria^a^				Fungi^b^					
	*Bs*	*Ec*	*Pa*	*Sa*	*Aa*	*Bc*	*Cl*	*Fo*	*Gg*	*Tb*
**1**	>256	256	256	>256	>256	8	32	8	>256	>256
**2**	>256	256	256	>256	>256	16	32	16	>256	>256
Ch^c^	2	1	4	2						
Pr^d^					16	8	16	8	4	8

a Bs, *Bacillus subtilis*; Ec, *Escherichia coli*; Pa, *Pseudomonas aeruginosa*; Sa, *Staphylococcus aureus*.

b Aa, *Alternaria alternata*; Bc, *Botrytis cinerea*; Cl, *Colletotrichum lagenarium*; Fo, *Fusarium oxysporum* f. sp. *nicotianae*; Gg, *Gaeumannomyces graminis* var. *graminis*; Tb, *Thielaviopsis basicola*.

c Ch, positive control, chloromycetin.

d Pr, positive control, prochloraz.

## Conclusion

In summary, overexpression of the global regulator Talae1 upregulated the production of SMs in an endophytic fungus *T. afroharzianum*, and two new polyketides (**1** and **2**) were isolated and identified from overexpression transformant. These results indicate that the global regulator Talae1 is involved in secondary metabolic regulation of *T. afroharzianum* and affects the biosynthesis of a series of antifungal polyketides. This study also demonstrates that genetic manipulation of the global regulator presents a promising approach for activating new SMs and improving the metabolic potential of biocontrol fungi.

## Data Availability Statement

The datasets presented in this study can be found in online repositories. The names of the repository/repositories and accession number(s) can be found in the article/Supplementary Material.

## Author Contributions

ZD conceived and designed the experiments, prepared the manuscript, and was involved in isolation of compounds. XW and H-MH performed genetic manipulation, strain fermentation, and extraction. F-DK contributed to determinate the structures of isolated compounds. Y-NZ and ML contributed to bioactivity assay. Z-PW and JH supervised the work and revised the manuscript. All authors contributed to the article and approved the submitted version.

### Conflict of Interest

The authors declare that the research was conducted in the absence of any commercial or financial relationships that could be construed as a potential conflict of interest.
