# Supplementary material for: Overexpression of Global Regulator Talae1 Leads to the Discovery of New Antifungal Polyketides From Endophytic Fungus Trichoderma afroharzianum
Source: Front Microbiol. 2020 Dec 23;11:622785. doi: 10.3389/fmicb.2020.622785 (PMC7785522; doi:10.3389/fmicb.2020.622785)
Supplement: Supplementary file 1 [file Data_Sheet_1.docx]

Supplementary Material

Overexpression of Global Regulator Talae1 Leads to the Discovery of New Antifungal Polyketides from Endophytic Fungus *Trichoderma afroharzianum*

**Zhuang Ding**^1*^**, Xiao Wang**^1^**, Fan-Dong Kong**^2^**, Hui-Ming Huang**^3^**, Yan-Na Zhao**^1^**, Min Liu**^1^**, Zheng-Ping Wang**^1^**, Jun Han**^1^

^1^ *Institute of BioPharmaceutical Research, Liaocheng University, Liaocheng, 252059, People’s Republic of China*

^2^ *Hainan Key Laboratory for Research and Development of Natural Product from Li Folk Medicine, Institute of Tropical Bioscience and Biotechnology, Chinese Academy of Tropical Agriculture Sciences, Haikou 571101, People’s Republic of China*

^3^ *School of Life Sciences, Liaocheng University, Liaocheng, 252059, People’s Republic of China*

*** Correspondence:**Zhuang Ding
dingzhuang@lcu.edu.cn

**TABLE OF CONTENT**

**Experimental procedures**

**1.** Theory and Calculation Details

**2.** RNA Extraction and Real-Time PCR Analysis

**Supplementary tables and figures**

**Table S1.** Primers used in this study

**Table S2.** B3LYP/6-31g(d) optimized lowest energy 3D conformers of compound **1**.

**Figure S1.** Phylogenetic tree analysis generated based on the sequences of tef1 (A) and rpb2 (B), which includes only species in the *Trichoderma harzianum* complex.

**Figure S2.** Map of the overexpression plasmid pZeo-talae1.

**Figure S3.** PCR verification of overexpression strain.

**Figure S4.** Quantitative RT-PCR analysis of *talae1* expression levels.

**Figure S5.** HRESI-MS spectrum of compound **1**.

**Figure S6.** ^1^H-NMR spectrum of compound **1** *(CD_3_OD)*.

**Figure S7.** ^13^C-NMR spectrum of compound **1** *(CD_3_OD)*.

**Figure S8.** ^13^C DEPT spectrum of compound **1** *(CD_3_OD)*.

**Figure S9.** HMQC spectrum of compound **1** *(CD_3_OD)*.

**Figure S10.** COSY spectrum of compound **1** *(CD_3_OD)*.

**Figure S11.** HMBC spectrum of compound **1** *(CD_3_OD)*.

**Figure S12.** HRESI-MS spectrum of compound **2**.

**Figure S13.** ^1^H-NMR spectrum of compound **2** *(CD_3_OD)*.

**Figure S14.** ^13^C-NMR spectrum of compound **2** *(CD_3_OD)*.

**Figure S15.** ^13^C DEPT spectrum of compound **2** *(CD_3_OD)*.

**Figure S16.** HMQC spectrum of compound **2** *(CD_3_OD)*.

**Figure S17.** COSY spectrum of compound **2** *(CD_3_OD)*.

**Figure S18.** HMBC spectrum of compound **2** *(CD_3_OD)*.

**Figure S19.** Normal (A) and Chiral HPLC (B) of compound **1**.

**Figure S20.** UV Spectra of compound **1**.

**Supplementary references**

**Experimental procedures**

**1.** Theory and Calculation Details

The calculations were performed by using the density functional theory (DFT) as carried out in the Gaussian 03.^S1^ The preliminary conformational distributions search was performed using Frog2 online version.^S2^ The conformers were further optimized at the B3LYP/6-31g(d) level, and 11 lowest electronic transitions were calculated (Table S2). Solvent effects of methanol solution were evaluated at the same DFT level by using the SCRF/PCM method.^S3^ TDDFT^S4^ at B3LYP/6-31G(d) was employed to calculate the electronic excitation energies and rotational strengths in methanol.

**2.** RNA Extraction and Real-Time PCR Analysis

The relative expression levels of *talae1* gene in the *OE::Talae1* and control strain were analyzed by real-time PCR. The transformant and control strain were cultivated in rice medium at 28°C. The mycelia of each strain were collected on the 10th day, and RNA was extracted from the mycelia using the EasyPure^®^ RNA kit for qRT-PCR (ER101, TransGen Biotech, China) following the manufacturer’s protocol. The quality of the RNA was checked by a NanoDrop 2000 nucleotide analyser (Thermo Scientific, Waltham, MA, USA). cDNA was synthesized using the TransScript® kit (AH341, TransGen Biotech, China). Real-time PCR was performed using a CFX96 Real-Time System (Bio-Rad, Hercules, CA, USA) using the TransStart^®^ kit (AQ132, TransGen Biotech, China). The PCR reaction system were 10 μL 2 × qPCR mix, 0.5 μL forward/reverse primer (10 μM), 1 μL template cDNA, and water to 20 μL. The PCR reaction conditions were 95 °C for 2 min, following by 45 cycles of 95 °C for 5 s, 60 °C for 15 s, 72 °C for 20 s. Three replicates of each cDNA sample were analyzed, and the average threshold cycle was calculated. Relative expression levels were calculated using the 2 ^−ΔΔCt^ method with the expression level of the *tef1* gene as the internal control. The primers used for real-time PCR are listed in Table S1.

**Supplementary tables and figures**

**Table S1.** Primers used in this study.

| **Primer name** | **Sequence 5'-3'** | **Function** |
| --- | --- | --- |
| Ta85012oF | GATGACGGCTGAGATTTCAC | amplification of *laeA*-like gene |
| Ta85012oR | TCTATACTTCTGTATGATGG |  |
| Ta85012iF | AAATtctagaATGTCGCTCAACGCTC |  |
| Ta85012iR | AAATgatatcTTACAGAGTGCAGTAG |  |
|  |  |  |
| Check-1 | GTTATCCCCTGATTCTGTGG | diagnostic PCR for overexpression strain |
| Check-2 | ATGTCGCTCAACGCTCGAAA |  |
| Check-3 | TTACAGAGTGCAGTAGGCGT |  |
| Check-4 | AGCAAAGATTGAATAAGGCG |  |
|  |  |  |
| RTtalea1F | CTGCTCGCTGGTTCAATC | Real Time-PCR |
| RTtalea1R | CCTTCTTGACTCTGTTGC |  |
| RTtef1F | TTCCTTCAAGTACGCTTG |  |
| RTtef1R | ATGACGGTGACATAGTAC |  |

**Table S2.** B3LYP/6-31g(d) optimized lowest energy 3D conformers of compound **1**.

| **No.** | **Boltzmann Distribution (%)** | **Lowest energy 3D conformer** |
| --- | --- | --- |
| **1** | 20.10 | 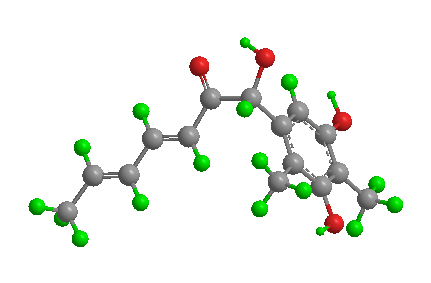 |
| **2** | 0.04 | 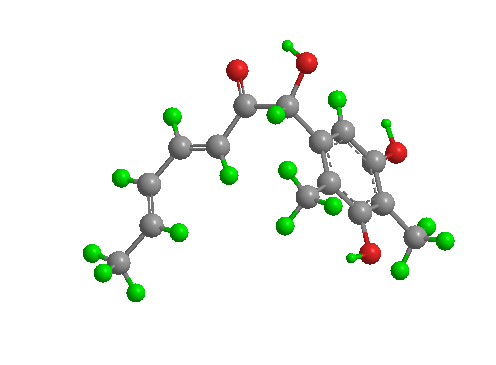 |
| **3** | 0.04 | 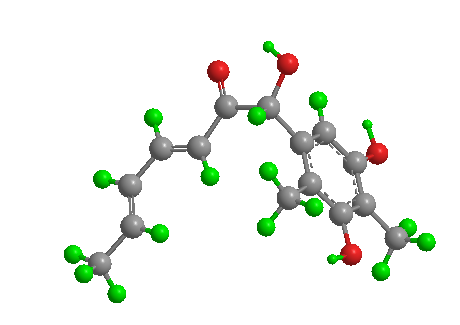 |
| **4** | 52.62 | 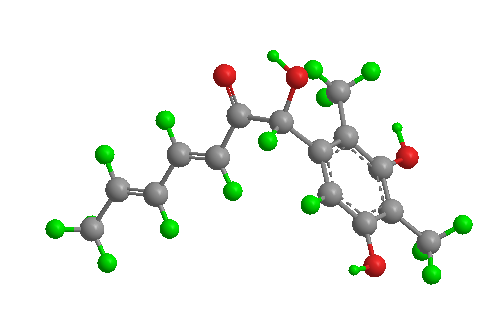 |
| **5** | 0.01 | 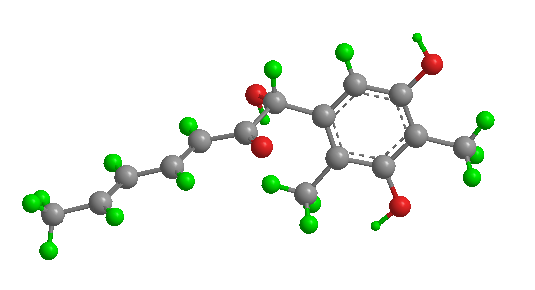 |
| **6** | 90.9 | 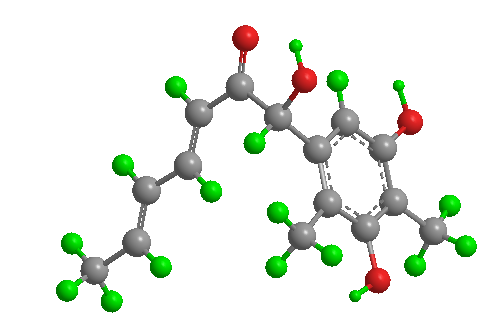 |
| **7** | 17.97 | 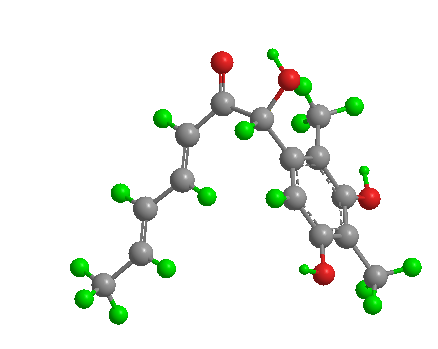 |
| **8** | 0.10 | 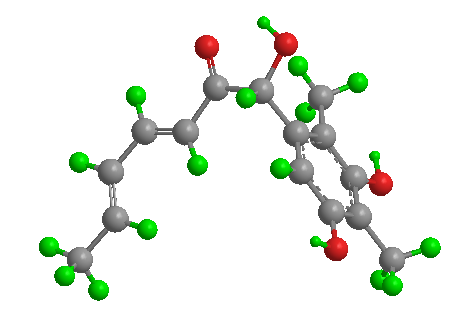 |
| **9** | 0.10 | 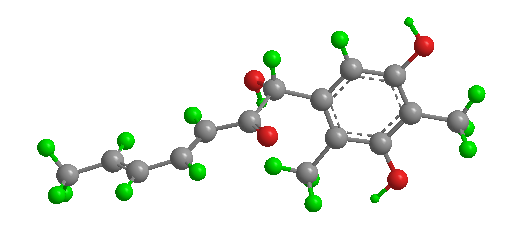 |
| **10** | 0.00 | 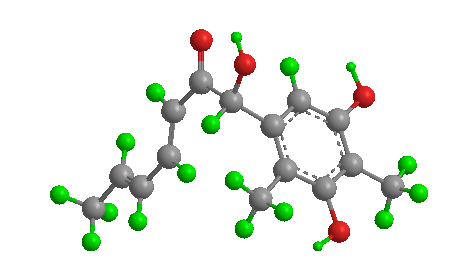 |
| **11** | 0.01 | 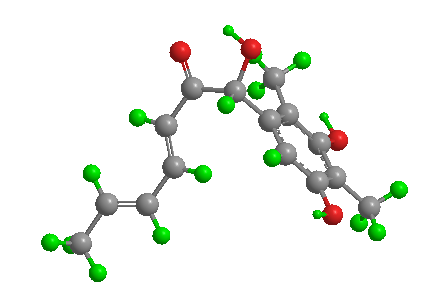 |


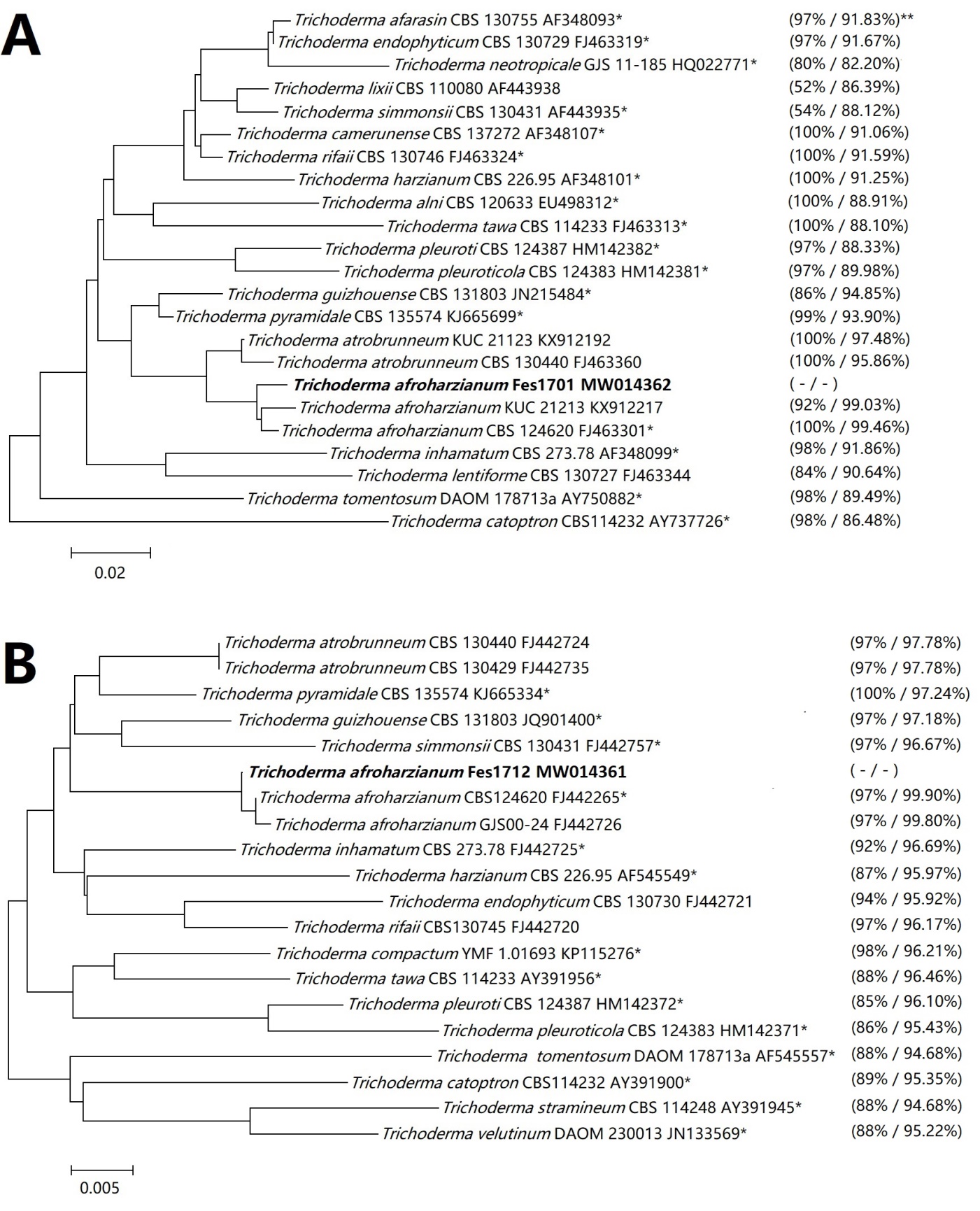


**Figure S1.** Phylogenetic tree analysis generated based on the sequences of *tef1* (A) and *rpb2* (B), which includes only species in the *Trichoderma harzianum* complex. Branch lengths are in proportion to distance. The * indicates a type culture. The ** indicates that the BLAST results (cover / identity percent) of *tef1* or *rpb2* sequence aligned with each *Trichoderma harzianum* complex.


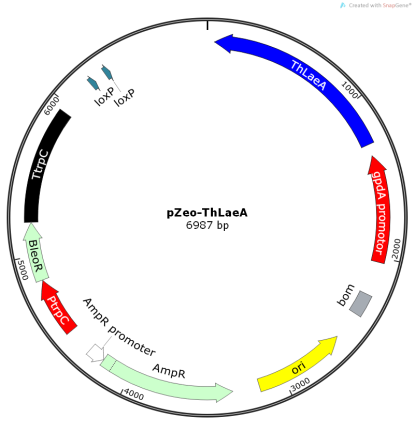


**Figure S2.** Map of the overexpression plasmid pZeo-talae1.

**Figure S3.** PCR verification of OE::Talae1 strain. (A) Schematic illustration of diagnostic PCR. Three pairs of primers were used for PCR verification of mutant genotype. The 2.9-kb fragment can be amplified from OE::Talae1 mutant using primers C1-C4, while only 1.7-kb fragment appears in control strain. The 2.4-kb and 1.7-kb fragments can be amplified from correct OE::Talae1 mutant using primers C1-C3 and C2-C4, respectively, but was absent in control strain. (B) Genotypical verificant of mutant by PCR. Note: Lane TG, using primers C1-C4; Lane 5F, using primers C1-C3; Lane 3F, using primers C2-C4. DNA Marker III (Tiangen Biotech Co., Ltd., Beijing, China) was used in 1% agarose gel electrophoresis.


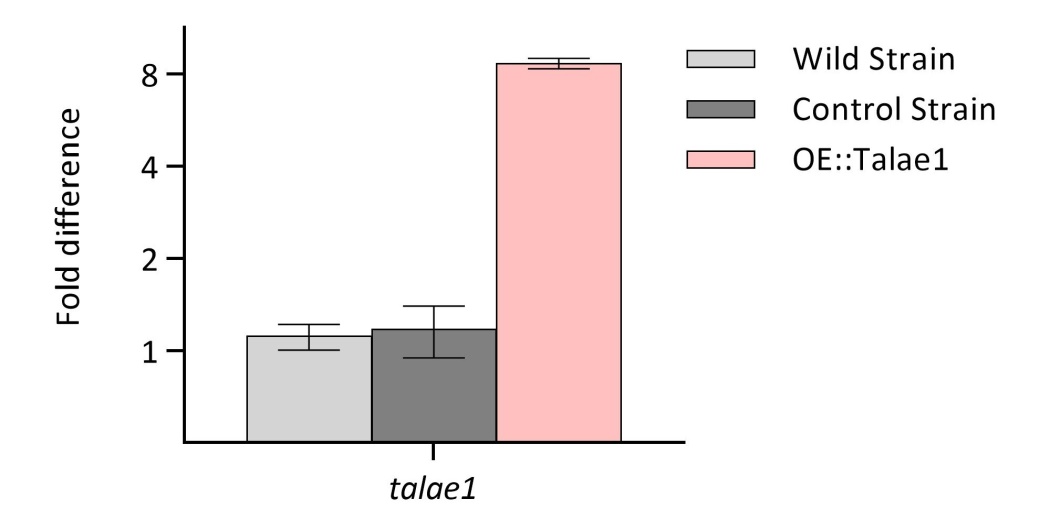


**Figure S4.** Quantitative RT-PCR analysis of *talae1* expression levels. The analysis for each strain was performed in triplicate. Data are shown as fold change relative to the first trial of the control strain.

**Figure S5.** HRESI-MS spectrum of compound **1**.


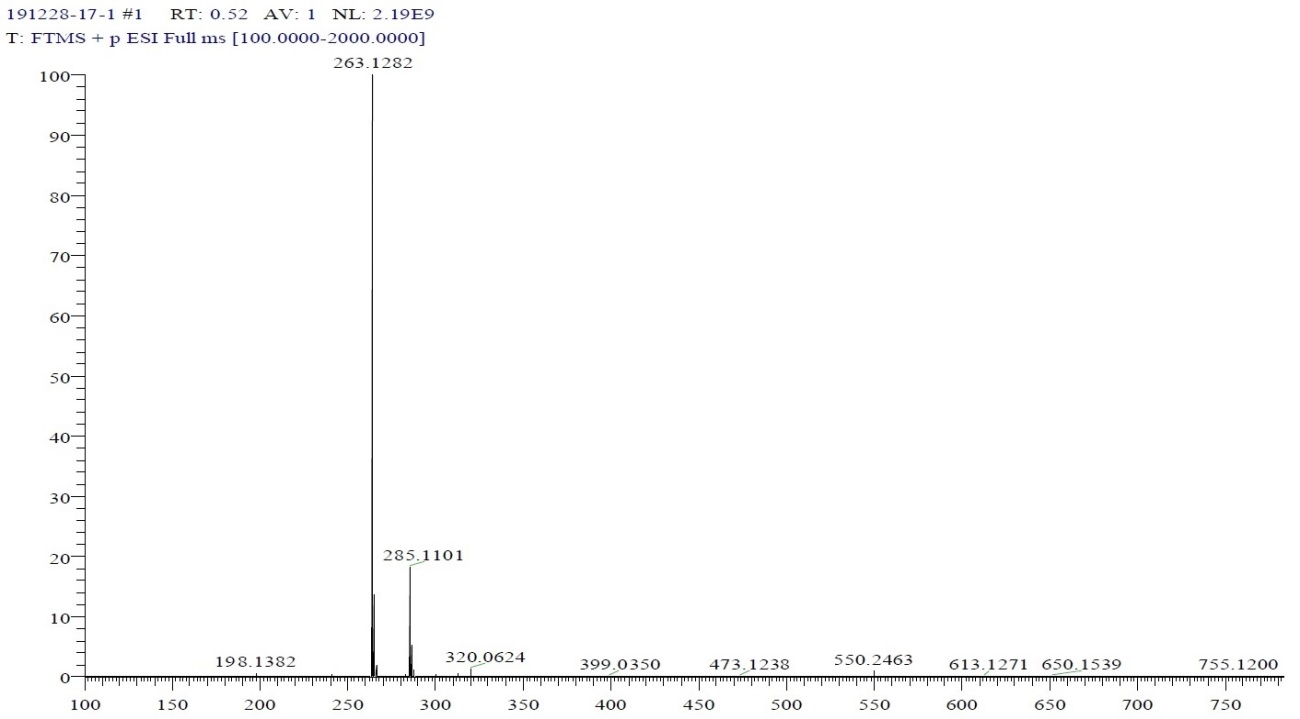


**Figure S6.** ^1^H-NMR spectrum of compound **1** *(CD_3_OD)*.

**Figure S7.** ^13^C-NMR spectrum of compound **1** *(CD_3_OD)*.

**Figure S8.** ^13^C DEPT spectrum of compound **1** *(CD_3_OD)*.

**Figure S9.** HMQC spectrum of compound **1** *(CD_3_OD)*.

**Figure S10.** COSY spectrum of compound **1** *(CD_3_OD)*.

**Figure S11.** HMBC spectrum of compound **1** *(CD_3_OD)*.

**Figure S12.** HRESI-MS spectrum of compound **2**.


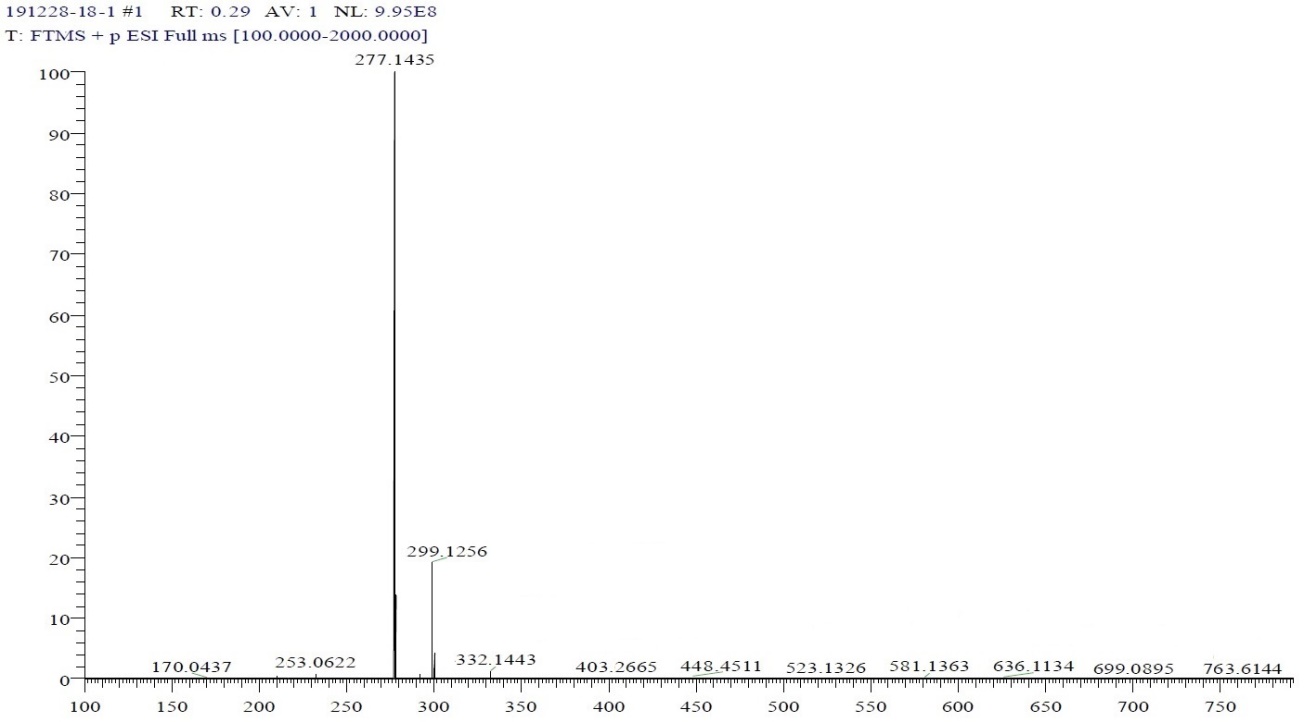


**Figure S13.** ^1^H-NMR spectrum of compound **2** *(CD_3_OD)*.

**Figure S14.** ^13^C-NMR spectrum of compound **2** *(CD_3_OD)*.

**Figure S15.** ^13^C DEPT spectrum of compound **2** *(CD_3_OD)*.

**Figure S16.** HMQC spectrum of compound **2** *(CD_3_OD)*.

**Figure S17.** COSY spectrum of compound **2** *(CD_3_OD)*.

**Figure S18.** HMBC spectrum of compound **2** *(CD_3_OD)*.

**Figure S19.** Normal (A) and Chiral HPLC (B) of compound **1**.

**
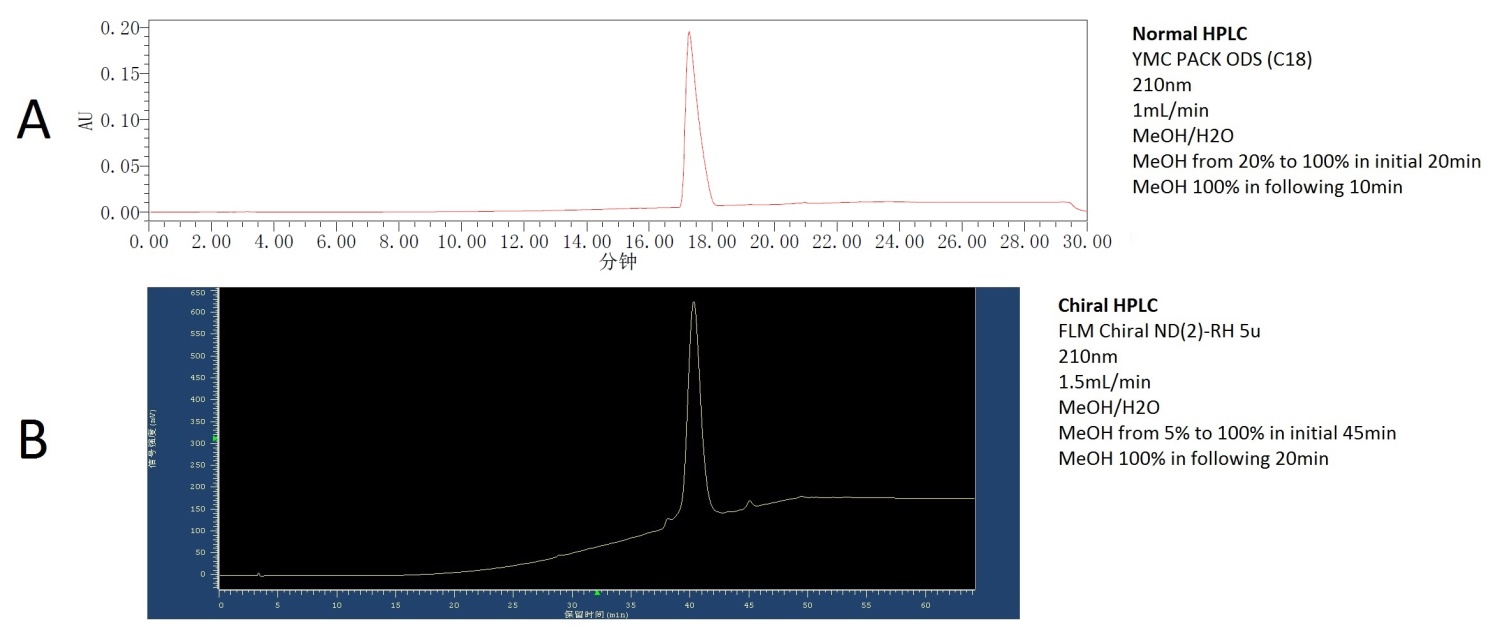
**

**Figure S20.** UV Spectra of compound **1**.

**
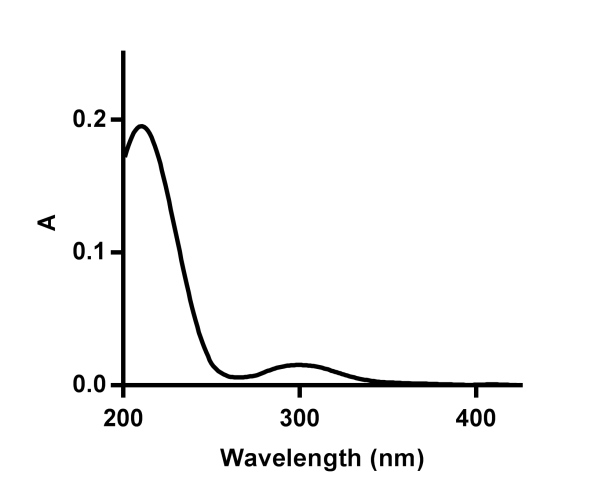
**

**Supplementary references**

(S1) Frisch, M. J., Trucks, G. W., Schlegel, H. B., Scuseria, G. E., Robb, M. A., Cheeseman, J. R., et al. (**2009**). *Gaussian 09, Revision A.1, Gaussian, Inc.*, Wallingford, CT.

(S2) Miteva, M. A., Guyon, F., Tuffery, P. (2010). *Nucleic Acids Res.* 38, 622–627.

(S3) Sai, C., Li, D., Xue, C., Wang, K., Hu, P., Pei, Y., et al. (2015). *Org. Lett.* 17, 4102–4105.

(S4) (a) Miertus, S., Tomasi, J. (1982). *Chem. Phys.* 65, 239–245. (b) Tomasi, J., Persico, M. (1994). *Chem. Rev.* 94, 2027–2094. (c) Cammi, R., Tomasi, J. (1995). *J. Comp. Chem.* 16, 1449–1458.
